# Computational Approaches and Drug Discovery: Where Are We Going?

**DOI:** 10.3390/molecules29050969

**Published:** 2024-02-22

**Authors:** Marco Tutone, Anna Maria Almerico

**Affiliations:** Dipartimento di Scienze e Tecnologie Biologiche Chimiche e Farmaceutiche (STEBICEF), Università degli Studi di Palermo, Via Archirafi 32, 90123 Palermo, Italy

Science is a point of view. Two centuries ago, Auguste Comte stated in 1830, “Any attempt to use mathematical methods in the study of chemical problems must be considered profoundly irrational and contrary to the spirit of chemistry…”. But just a few years later (1888), Gay-Lussac changed this perspective: “…perhaps we are not too far from the moment when we will be able to treat the heart of chemical phenomena with computation…”. The efforts to conjugate chemistry, mathematics, and computers were awarded the Nobel Prize in Chemistry in 1998, which was divided equally between Walter Kohn “for his development of the density-functional theory” and John A. Pople “for his development of computational methods in quantum chemistry”. They developed improved energy calculations on molecules and other multi-atom systems, enabling the chemists to perform calculations on systems during reactive encounters. These findings gave a better understanding of chemical dynamics and allowed for predictions regarding the course of chemical reactions [1]. Computational approaches rose to prominence again in 2013, when the Royal Swedish Academy of Science announced that the winners of the Nobel Prize in Chemistry were Martin Karplus, Michael Levitt, and Arieh Warshel for their studies “on the development of multiscale models for complex chemical systems”. Starting in 1970, the three scientists laid the foundation for programs used worldwide today to understand chemical processes. Karplus, Levitt, and Warshel developed equations that allow chemical processes to be simulated and even predict the outcome of reactions before carrying them out. Their equations are used every day in industrial chemistry and drug development. With progress and therefore the creation of computers with greater computing power, it has been possible to avoid idle experiments and finely regulate the processes that are desired to be achieved in a much shorter time. But what they have achieved goes much further; their research is at the basis of the birth of so-called target therapy, i.e., all those treatments in which specific targets are targeted, such as tumors. Furthermore, knowing the three-dimensional characteristics of some molecules in nature has allowed scientists to develop much more efficient analogs. These three new Nobel Prize winners managed to combine classical and quantum physics in the description of interactions between molecules.

Computational approaches used in the early stages of drug discovery depend on the constant increase in hardware performance. Moore’s Law [2], which states that computer power doubles every 24 months, gives an empirical projection of historical trends in hardware. Even though the miniaturization of chips will make Moore’s law obsolete by 2036, it is still possible to observe some advances in hardware performance due to the increasing use of GPUs, parallel CPUs, and the inclusion of new technologies [3]. This progress in hardware contributes to the expanding role of computational methods in searching for new drugs. This impact on drug discovery occurs by accelerating virtual screening initiatives, speeding up CPU-demanding simulations (e.g., molecular dynamics), and combining multiple steps of drug discovery in an optimized workflow. As a demonstration of this, the actual combinations of software and hardware allow the evaluation of several million compounds/day. This number could reach even billions, as stated by the researchers at the Oak Ridge National Laboratory working on the SUMMIT supercomputer, which was recently exploited for an ultra-large GPU-accelerated virtual screening against the SARS-CoV-2 main protease [4,5]

The chemical space forming the drug-like environment (10^60^ compounds) is probably the major challenge in the drug discovery field [6]. Medicinal chemists can overcome this limitation by bringing the hit selection problem from a wet lab setup to a virtual environment, such as HTVS. Without forgetting several examples of reverse virtual screening where a single compound is screened against thousands of targets [7,8].

In the last decade, the evolution of machine learning and deep learning methods focused on drug discovery [9], and on another front, the increase in protein-ligand data with over 200,000 structures in the protein data bank (PDB) enlarges the chances for drug discovery projects. Moreover, databases providing receptor–ligand affinity data fully integrated with structural data create an ideal scenario to address the early discovery phases both in academia and industry [10,11,12].

In this second Special Issue, we continued to collect [13] original research articles and reviews covering all the aspects of computational approaches applied to drug discovery. Virtual screenings remain of large use in the early phase of drug discovery and are applied to several different approaches. Jokinen and colleagues employed molecular docking and pharmacophore modeling in a VS campaign to identify retinoic acid-related orphan receptor γt modulators. Twenty-eight compounds were selected for in vitro testing, and eight showed low micromolar inhibitory activity, generating a hit rate of ~29% (Contribution 1). Haque and colleagues described the design and development of seventeen pyrimidine-clubbed benzimidazole derivatives as potential dihydrofolate reductase (DHFR) inhibitors that were filtered through ADMET and drug-likeness profiles before carrying out docking calculations. Their in vitro tests confirmed two compounds effective against all the bacterial and fungal strains selected (Contribution 2). Detroja and Samson performed a VS for FDA-approved drugs that selectively inhibit Arginase 1 and 2 by using docking and molecular mechanics energy calculations. Candesartan, ibersartan, codeine, metformin, and isavuconazole qualified as suitable candidates for the development of potential arginase inhibitors, even though in vitro and in vivo studies are required to characterize the effective ligand binding (Contribution 3). Ebenezer and colleagues performed an induced-fit (IFD) docking study on the ZINC library, identifying two promising candidates as potential norovirus inhibitors (Contribution 4).

Molecular dynamics is the approach of choice when a computational medicinal chemist looks for accuracy and the interpretation of the mechanism of action [8,14]. In this Special Issue, Conrad and colleagues tried to unveil the influence of different histamine tautomers (e.g., τ-tautomer and π-tautomer) and charge states (mono- vs. di-cationic) on the interaction with the ternary histamine–H1R–Gq complex by means of atomistic molecular dynamics (Contribution 5). Altharawi tried to interpret the mechanism of action of three compounds selected by docking and energy calculations as antiparasitic drugs that can block *Toxoplasma gondii* ME49 TgAPN2 (Contribution 6). The use of molecular dynamics and molecular mechanics energy calculation was exploited by Tiwari and colleagues, who showed the role of pre- and pro-vitamin D of mushrooms against Mpro and PLpro proteases of SARS-CoV2 (Contribution 7). Ali and colleagues screened traditional Chinese medicine’s natural compounds as myostatin (MSTN) inhibitors. Molecular dynamics of the most promising compound helped to understand its therapeutic potential (Contribution 8).

Moreover, computational approaches, such as QSAR, proved useful in providing a mechanistic interpretation of structure–activity relationships for the further development of active compounds. In this Special Issue, Bernal and Schmidt reported various QSAR models to explain and predict the antileishmanial activity of a series of dyhidrobenzofurans. They stated that the best-performing and robust 3D-QSAR model can guide the decision making of new antileishmanial compounds before synthesis (Contribution 9).

Three reviews were also collected in this Special Issue. Bassani and Moro reported the state-of-the-art computer-aided drug design methods, focusing on their application in different scenarios of pharmaceutical and biological interest and highlighting their potential and weaknesses (Contribution 10). Dulsat and colleagues compared eighteen free web servers capable of predicting ADMET properties and analyzed their advantages and disadvantages (Contribution 11).

Last but not least, Klupt and Jia presented an overview of eEF2K-related drug discovery efforts dating from the 1990s to more recent in vivo studies in rat models and their view regarding the future of eEF2K drug discovery (Contribution 12).

We express our deep gratitude again to all the contributors to this Special Issue for their commitment, hard work, and outstanding papers. We also thank all the reviewers involved in the manuscript revisions for their unpaid contributions to improve any aspects of the submitted works. We think that these manuscripts could contribute to the improvement of the drug discovery field.
